# Genetic but No Phenotypic Associations between Biocide Tolerance and Antibiotic Resistance in *Escherichia coli* from German Broiler Fattening Farms

**DOI:** 10.3390/microorganisms9030651

**Published:** 2021-03-21

**Authors:** Alice Roedel, Szilvia Vincze, Michaela Projahn, Uwe Roesler, Caroline Robé, Jens Andre Hammerl, Matthias Noll, Sascha Al Dahouk, Ralf Dieckmann

**Affiliations:** 1 German Federal Institute for Risk Assessment, Max-Dohrn-Str. 8-10, 10589 Berlin, Germany; alice_roedel@gmx.de (A.R.); Szilvia.Vincze@bfr.bund.de (S.V.); Michaela.Projahn@bfr.bund.de (M.P.); Jens-Andre.Hammerl@bfr.bund.de (J.A.H.); Sascha.Al-Dahouk@gmx.de (S.A.D.); 2 Institute for Animal Hygiene and Environmental Health, Freie Universität Berlin, Robert-von-Ostertag Str. 7-13, 14163 Berlin, Germany; Uwe.Roesler@fu-berlin.de (U.R.); caroline.robe@fu-berlin.de (C.R.); 3 Institute for Bioanalysis, University of Applied Sciences and Arts, Friedrich-Streib-Str. 2, 96450 Coburg, Germany; matthias.noll@hs-coburg.de

**Keywords:** *Escherichia coli*, biocide tolerance, antibiotic resistance, biocide determinants, virulence, food safety

## Abstract

Biocides are frequently applied as disinfectants in animal husbandry to prevent the transmission of drug-resistant bacteria and to control zoonotic diseases. Concerns have been raised, that their use may contribute to the selection and persistence of antimicrobial-resistant bacteria. Especially, extended-spectrum β-lactamase- and AmpC β-lactamase-producing *Escherichia coli* have become a global health threat. In our study, 29 ESBL-/AmpC-producing and 64 NON-ESBL-/AmpC-producing *E.* *coli* isolates from three German broiler fattening farms collected in 2016 following regular cleaning and disinfection were phylogenetically characterized by whole genome sequencing, analyzed for phylogenetic distribution of virulence-associated genes, and screened for determinants of and associations between biocide tolerance and antibiotic resistance. Of the 30 known and two unknown sequence types detected, ST117 and ST297 were the most common genotypes. These STs are recognized worldwide as pandemic lineages causing disease in humans and poultry. Virulence determinants associated with extraintestinal pathogenic *E.* *coli* showed variable phylogenetic distribution patterns. Isolates with reduced biocide susceptibility were rarely found on the tested farms. Nine isolates displayed elevated MICs and/or MBCs of formaldehyde, chlorocresol, peroxyacetic acid, or benzalkonium chloride. Antibiotic resistance to ampicillin, trimethoprim, and sulfamethoxazole was most prevalent. The majority of ESBL-/AmpC-producing isolates carried *bla*_CTX-M_ (55%) or *bla*_CMY-2_ (24%) genes. Phenotypic biocide tolerance and antibiotic resistance were not interlinked. However, biocide and metal resistance determinants were found on mobile genetic elements together with antibiotic resistance genes raising concerns that biocides used in the food industry may lead to selection pressure for strains carrying acquired resistance determinants to different antimicrobials.

## 1. Introduction

*Escherichia coli* is a gram-negative, non-sporulating facultative anaerobe, a widespread gut commensal of vertebrates, and a versatile pathogen [1]. Pathogenic *E. coli* are categorized as intestinal pathogenic (InPEC) or extraintestinal pathogenic *E. coli* (ExPEC) [2]. The latter colonize the gut of healthy hosts without causing disease but by entering extraintestinal sites ExPEC can lead to urinary tract infections, meningitis, skin infections, or sepsis [3]. In addition to affecting humans, avian pathogenic *E. coli* (APEC), the avian pathotype of ExPEC, causes severe economic losses to the poultry industry and may represent a zoonotic risk [4]. Multidrug-resistant bacteria (particularly those producing extended-spectrum ß-lactamases (ESBL) and/or AmpC ß-lactamases (AmpC)) are a growing threat to food safety [5,6]. ESBL-/AmpC-producing *E. coli* from healthy hosts were classified as commensal strains but recent investigations indicated that they also show characteristics of ExPEC or ExPEC-like strains [3,7]. Humans can be exposed to ESBL-/AmpC-producing pathogens via human-to-human transmission, food, animal, and environmental sources [8]. A high prevalence of ESBL-/AmpC-producing Enterobacteriaceae was previously demonstrated on broiler farms [9,10,11]. Recent studies suggested that contaminated broiler chicken farms might play an important role in the transmission of ESBL-/AmpC-producing Enterobacteriaceae into the environment [12,13]. Luyckx et al. detected *E. coli* in broiler houses following hygiene measures, highlighting drain holes or floor cracks as critical locations for cleaning and disinfection (C&D) [14,15]. Biocides like quaternary ammonium compounds (QACs), aldehydes, oxidizing agents, organic acids, and cresols are widely used in animal husbandry and food processing plants to prevent microbial growth. However, concerns have been raised that the continued exposure to biocides in industrial settings including food production environments may trigger mechanisms that alter both biocide and antibiotic susceptibility and select for antimicrobial-resistant strains [16,17]. *E. coli* uses multiple pathways to overcome environmental stresses. Acid stress, for instance, is counteracted by a range of physiological, metabolic, and proton-consuming acid resistance mechanisms [18]. Biocide tolerance is a multifactorial process and can include several mechanisms such as target modification [19], biofilm formation [20], changes of cell envelope permeability [21], or the activity of efflux pumps [22]. Proteins involved in tolerance to quaternary ammonium compounds (QACs) include members of the small multidrug resistance (SMR) efflux family such as SugE(c), SugE(p), EmrE, YdgE/YdgF, QacE, QacE∆1, QacF, QacG, QacH, and QacI as well as members of the major facilitator superfamily (MFS) such as MdfA [23,24,25,26].

So far, laboratory methods to investigate biocide susceptibility are not standardized [27,28] and to the best of our knowledge, only one study evaluated epidemiological cutoffs (ECOFFs) for *E. coli* to a limited set of biocidal compounds [29]. As little is known about the link between biocide selection pressure and antibiotic resistance in *E. coli* field isolates in Germany we aimed to characterize a commensal *E. coli* study population including ESBL-/AmpC-producing and NON-ESBL-/AmpC-producing *E. coli* from broiler fattening farms following cleaning and disinfection. Because of the widespread use of disinfectants in hygiene processes, we assumed a high selective pressure in the investigated farm environment. We tested susceptibilities to seven biocides frequently used in farm hygiene and to antibiotics relevant for human and veterinary medicine. In addition, we characterized the genetic diversity of the *E. coli* strains including ExPEC associated virulence genes, and looked for associations between biocide tolerance, antibiotic resistance, and the presence of putative genetic determinants of antimicrobial resistance.

## 2. Materials and Methods 

### 2.1. E. coli Isolates

A panel of 93 *E. coli* isolates collected in 2016 from three broiler fattening farms after cleaning and disinfection measures were investigated (Table S1). The isolates originated from surfaces of grounds, walls, and equipment such as air inlets, drains, door handles, tractors (for food and litter), electric cables, feeding and drinking troughs from four barns. *E. coli* were isolated from swab samples on MacConkey agar with and without cefotaxime. Species identification and differentiation of ESBL-/AmpC-producing *E. coli* were performed as previously described [30]. In brief, MALDI-TOF MS (Bruker Daltonics, Bremen, Germany) was applied to suspicious isolates for species identification. Beta-lactamase genes *bla*_CTX-M_, *bla*_SHV_, *bla*_TEM_, and CIT-type pAmpC genes were identified using a multiplex real-time PCR [31] as well as Sanger sequencing [30]. Isolates were selected from different sources to obtain a highly diverse study population including ESBL-/AmpC- and NON-ESBL-/AmpC-producing *E. coli* (farm 1: barn 1, *n* = 27 including 13 AmpC-producing *E. coli*; barn 2, *n* = 15 including five ESBL-producing *E. coli*; farm 2: barn 3, *n* = 21 including three ESBL-producing *E. coli*; farm 3: barn 4, *n* = 30 including eight ESBL-producing *E. coli*). C&D protocols applied in the barns comprised dry cleaning, wet cleaning, and two disinfection steps. During dry cleaning, bedding and feed were removed. For wet cleaning all-purpose cleaners were used. Disinfection was carried out using formaldehyde-based disinfectants followed by either chlorocresol-based disinfectants (barns 1 and 4) or lime solutions (barns 2 and 3).

### 2.2. Whole Genome Sequencing

*E. coli* isolates were cultivated on sheep blood agar. A single colony was transferred into Miller’s lysogeny broth (LB) (Merck KGaA, Darmstadt, Germany) and incubated at 37 °C for 19 ± 1 h with shaking at 150 rpm. DNA was extracted using the PureLink Genomic DNA Mini Kit (Invitrogen, Carlsbad, CA, USA). Whole-genome sequencing (WGS) libraries were prepared with the Nextera XT DNA Sample Preparation Kit (Illumina, San Diego, CA, USA) according to the manufacturer’s protocol. Paired-end sequencing (2 × 301 cycles) was performed using the MiSeq Reagent v3 600-cycle Kit (Illumina) on an Illumina MiSeq benchtop sequencer. Raw fastq data were trimmed and assembled using the AQUAMIS pipeline (https://gitlab.com/bfr_bioinformatics/AQUAMIS (accessed on 9 July 2018)) based on trimmomatic (version 0.36.), fastp (version 0.19.5), unicycler (version 0.4.4), spades (version 3.11.1), pilon (version 1.22), mash (version 2.1), and quast (version 4.6.3).

### 2.3. Phylogenetic Analysis

For phylogenetic analysis, multilocus-sequence typing (MLST) was performed using WGS data. The classical MLST scheme defined by alleles of seven housekeeping genes (*adk*, *fumC*, *gyrB*, *icd*, *mdh*, *purA*, and *recA*, database hosted at the University of Warwick) was applied. MLST types were determined using the MLST 2.0 webtool of the Center for Genomic Epidemiology (http://www.genomicepidemiology.org (accessed on 6 October 2018)) [32]. For phylogroup assignment, a multiplex PCR was conducted as described previously [33] with minor modifications. The total reaction mixture of 25 µL contained 0.2 µM of each primer (except for TspE4C2.1b (0.4 µM) and TspE4C2.2b (0.4 µM)), 12.5 µL of DreamTaq Green PCR Mastermix (Thermo Fisher Scientific, Schwerte, Germany), 5 µL of PCR Water and 2.5 µL of the template DNA. An initial denaturation step of 3 min at 94 °C was followed by 33 PCR cycles with 30 s of denaturation at 94 °C, primer binding for 30 s at 57 °C, and 1 min of elongation at 72 °C, as well as a final elongation step of 5 min at 72 °C. Isolates belonging to phylogroups A and C or E and D were not further differentiated and assigned to phylogroup A/C or E/D, respectively. Furthermore, we determined genetic relatedness between *E.coli* isolates with ParSNP v1.0 [34]. The maximum-likelihood tree was calculated by FastTree2 [35] and visualized with EMBL interactive tree of life, iTOL v4 (https://itol.embl.de/, accessed on 20 September 2019).

### 2.4. Biocide Susceptibility Testing

#### 2.4.1. Biocides

Susceptibility of the *E. coli* isolates was tested against the two biocides formaldehyde (FA, Carl Roth, Karlsruhe, Germany) and chlorocresol (p-chloro-m-cresol, PCMC, Merck KGaA) used for C&D on the farms under study and five biocides commonly applied in farm hygiene, namely the quaternary ammonium compounds benzalkonium chloride (BAC, Sigma Aldrich, Steinheim, Germany) and didecyldimethylammonium chloride (DDAC, Merck KGaA), hydrogen peroxide (HP, Carl Roth), peroxyacetic acid (PAA, VWR, Dresden, Germany), and acetic acid (AA, Carl Roth). Biocides were serially diluted in 2-fold steps just before the experiment using standardized hard water as defined in EN 1276. The following final concentration ranges were tested: 320 to 5 mg/L BAC, 40 to 0.3 mg/L DDAC, 640 to 5 mg/L FA, 1024 to 8 mg/L HP, 2000 to 16 mg/L PAA, 16,384 to 128 mg/L AA, and 4000 to 63 mg/L PCMC.

#### 2.4.2. Minimum Inhibitory Concentration (MIC)

Biocide MICs were determined using broth microdilution. Overnight cultures grown on tryptic soy agar (TSA; Merck KGaA) were adjusted to about 10^6^ CFU/mL in 2-fold concentrated tryptic soy broth (TSB; Merck KGaA). In 96-well microtiter plates (Greiner Bio-One, Frickenhausen, Germany), 50 µL of the bacterial suspension was added to 50 µL of the double-concentrated biocide solution. Plates were incubated at 37 °C for 20 ± 2 h. Optical density at 595 nm (OD595) was measured after 5 s of shaking using the Mithras2 multimode reader (Berthold Technologies, Bad Wildbad, Germany; Software MikroWin 2010 v5.18, German UI). Bacterial growth was compared to a negative control (microtiter well containing biocide solution and tryptic soy broth, Thermo Fisher Scientific) and a ΔOD_595 nm_ of 0.08 was applied as the cut-off value. The MIC was defined as the lowest concentration of a biocide at which no growth was observed. Three independent experiments were performed on different days and the median was considered as the final MIC.

#### 2.4.3. Minimum Bactericidal Concentration (MBC)

The MBC of each strain and biocide was determined by broth microdilution according to Knapp et al., with minor modifications [28]. Dey-Engley neutralizing broth (Sigma-Aldrich) was used to quench biocidal effects for MBC testing. Neutralizer efficacy and toxicity were tested before [36]. The MBC was defined as the lowest concentration of a biocide, which revealed no visible colonies after subculture on tryptic soy agar (TSA, Thermo Fisher Scientific). The reference strain *E. coli* ATCC 25922 was used as internal quality control in both MIC and MBC tests and showed comparable results throughout the experiments.

#### 2.4.4. Determination of MIC_95_/MBC_95_

To distinguish between biocide susceptible isolates and isolates with reduced susceptibility, the MIC (or MBC) that encompassed 95% of all MIC (or MBC) values in the distribution was designated as MIC_95_ (or MBC_95_).

### 2.5. Antibiotic Susceptibility Testing

Antibiotic susceptibility was determined by broth microdilution using the Sensititre system with EUVSEC/EUVSEC2 plates (Thermo Fisher Scientific) in concordance with the decision 2013/652/EU of the European Union. The following antimicrobial substances were used: Ampicillin, AMP; Azithromycin, AZI; Cefepime, FEP; Cefoxitin, FOX; Ceftazidime, TAZ; Cefotaxime, FOT; Cefotaxime/Clavulanic acid, F/C; Ceftazidime/Clavulanic acid, T/C; Chloramphenicol, CHL; Ciprofloxacin, CIP; Colistin, COL; Ertapenem, ETP; Gentamicin, GEN; Imipenem, IMI; Meropenem, MERO; Nalidixic acid, NAL; Sulfamethoxazole, SMX; Temocillin, TRM; Tetracycline, TET; Tigecycline, TGC; Trimethoprim, TMP. We followed CLSI guidelines and defined resistance using epidemiological cut-offs according to EUCAST.

### 2.6. Statistical Analysis

Spearman rank coefficients (Rho) were calculated to investigate the correlation of MICs or MBCs between tested biocides and antibiotics using SPSS (IBM SPSS Statistics, Version 21, IBM corp., Armonk, NY, USA). Data were tested for normal distribution by the Kolmogorov-Smirnov test. For comparative analysis between two groups of isolates (e.g., ESBL-/AmpC- versus NON-ESBL-/AmpC-producing isolates) the Mann-Whitney-test was applied. Statistically significant differences between antimicrobial resistance or distribution of virulence determinants in different genetic lineages were tested using the chi2 test and Fisher’s exact test. *p*-values < 0.05 were considered to be significant.

### 2.7. In Silico Screening for Biocide and Metal Tolerance Determinants at Protein Level

WGS data of the *E. coli* isolates under study were screened for the presence of 753 experimentally confirmed biocide- and metal-resistance proteins recorded in the BacMet database [37] (Antibacterial Biocide and Metal Resistance Genes database; http://bacmet.biomedicine.gu.se/, BacMet version 2, last updated on 9 December 2017, accessed on 5 December 2018) as described before [38].

### 2.8. Detection of Biocide Tolerance and Virulence Determinants at Nucleotide Level

The presence of genes conferring biocide tolerance was determined as previously described [38]. The genomes of all isolates were screened for genes encoding for small multidrug resistance (SMR) transporters, i.e., *qacEΔ1, qacE*, *qacF*, *qacH*, *qacI*, *qacG*, *emrE*, *sugE(c)*, *sugE(p)*, *ydgE*, *ydgF*, and for the multidrug efflux pump gene *mdfA* of the major facilitator superfamily (MFS). In addition, we screened for genes involved in formaldehyde and acid tolerance. An overview of the investigated genes and corresponding accession numbers is given in Table S2. A minimum sequence identity (%ID) threshold of 80% and a minimum length of 80% of the target gene were defined for the detection of biocide determinants except for *qacEΔ1* and *qacE* (100%ID and 100% minimum length).

In addition, we screened for the presence of 49 virulence genes typically associated with ExPEC including fitness factors that are found in pathogenic and commensal strains (Table S2). Virulence-associated genes (VAGs) were chosen from public databases contained in the *E. coli* functional genotyping plugin (version 1.01) of Bionumerics or from previously published reports [7,39,40]. A minimum sequence identity (%ID) threshold of 90% and a minimum length of 60% of the target gene were used for the identification of VAGs.

### 2.9. Identification of Antibiotic Resistance Genes

Acquired antibiotic resistance determinants and chromosomal mutations leading to antibiotic resistance were identified using ResFinder 3.0 (Center for Genomic Epidemiology, http://www.genomicepidemiology.org, accessed on 11 January 2019 [41]).

### 2.10. Accession Numbers of Whole-Genome Sequences

Genome sequence data of the strains under study have been deposited at the National Center for Biotechnology Information database (https://www.ncbi.nlm.nih.gov/, accessed on 9 March 2021)) under accession numbers JAFMWT000000000-JAFMVF000000000 (see Table S1).

## 3. Results

### 3.1. Phylogenetic Diversity and Virulence-Associated Genes

**Figure 1:**
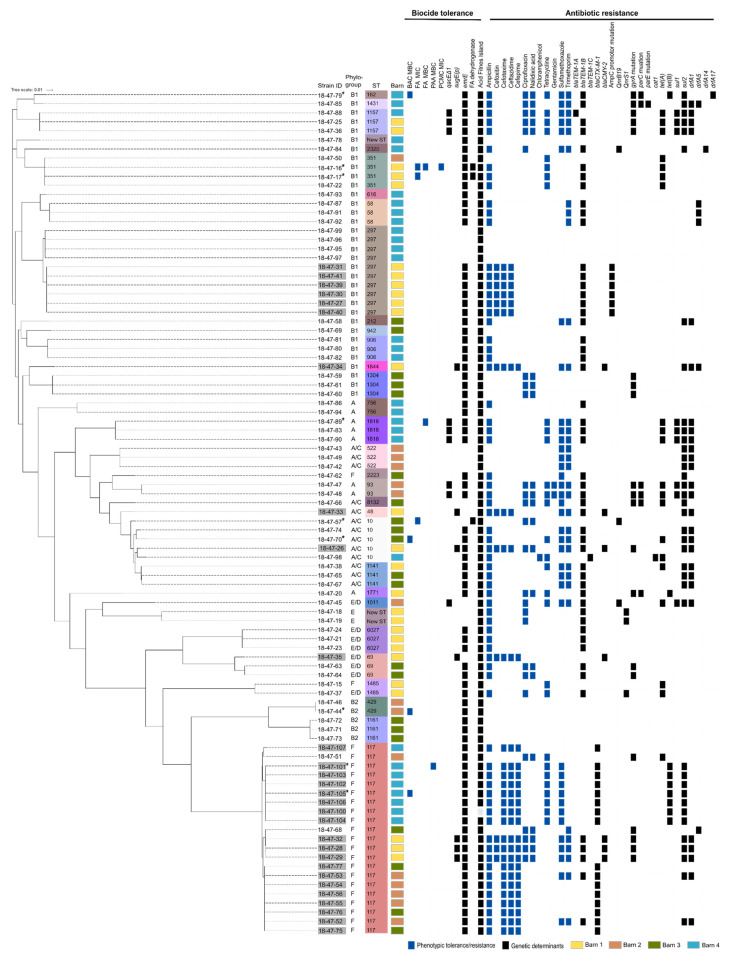
Phylogenetic tree of 93 *E. coli* isolates from broiler fattening farms including their phenotypic biocide tolerance and antibiotic resistance as well as the distribution of biocide tolerance and antibiotic resistance-conferring genes. An asterisk marks biocide tolerant strains. Reduced susceptibility to biocides and antibiotic resistance are indicated for each isolate as blue squares, tolerance, and resistance-conferring genes as black squares. Further information on ESBL-/AmpC-producing *E. coli* phenotype (grey shaded strain ID) and multilocus sequence type (ST) are provided. The affiliation to different barns are highlighted in yellow (barn 1), orange (barn 2), green (barn 3), and blue (barn 4). BAC = Benzalkonium chloride, FA = Formaldehyde, PCMC = Chlorocresol (p-chloro-m-cresol).

PCR-based phylotyping of the 93 *E. coli* isolates revealed seven different banding patterns associated with phylogroups A (*n* = 8), A/C (*n* = 13), B1 (*n* = 34), B2 (*n* = 5), E (*n* = 2), E/D (*n* = 7), and F (*n* = 24). *E. coli* isolates belonged to 30 known and two unknown multilocus sequence types (STs). The most prevalent STs were ST117 (*n* = 21; two NON-ESBL-/AmpC-producing *E. coli* from barns 2 and 3, 19 ESBL-/AmpC-producing *E. coli* from all barns) and ST297 (*n* = 10; six AmpC-producing *E. coli* from barn 1, and four NON-ESBL-/AmpC-producing *E. coli* from barn 4) (Figure 1).

Up to 27 ExVAGs (VAGs associated with ExPEC) (55%) were detected in ST117 strains (phylogroup F), up to 23 ExVAGs (47%) in ST429 (phylogroup B2), and up to 20 ExVAGs (41%) in ST69 (phylogroup E/D) (Table S1). All isolates were positive for *fimH* (type 1 fimbriae), *feoB* (ferrous iron transporter, protein B), and *ompA* (outer membrane protein A). The *iss* (increased serum survival protein) and *fimA* (type 1 fimbriae) genes were present in 77 (83%) and 74 (80%) isolates, respectively. Twenty-one VAGs were significantly associated with phylogroup F. Certain genetic determinants such as *papC*, *papEF*, *papG-allele II* (P fimbriae formation), *ireA* (iron-responsive element), and *hlyE* (hemolysin E) exclusively occurred in isolates belonging to ST117 of phylogroup F, whereas *vat* (vacuolating autotransporter toxin) was present in ST429 (phylogroup B2) and some ST117 (phylogroup F) isolates. Iron capture systems were frequently represented in the genomes, but the number of encoding genes varied considerably among isolates from 1 to 11. Iron uptake systems were most prevalent in ST117 and ST429 isolates.

### 3.2. Susceptibility to Biocides

**Figure 2:**
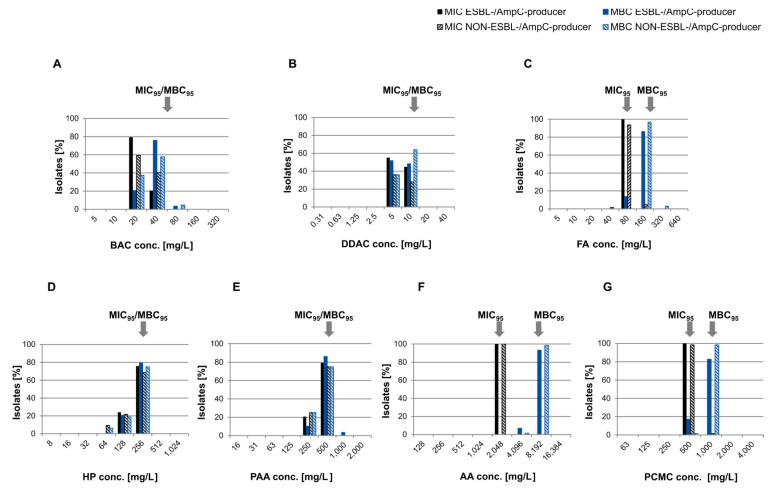
MIC and MBC distributions of ESBL-/AmpC-producing and NON-ESBL-/AmpC-producing *E. coli* isolates for common biocides used in farm hygiene. Black bars = MIC ESBL-/AmpC-producing *E. coli*, black striped = MIC NON-ESBL-/AmpC-producing *E. coli*, blue bars = MBC ESBL-/AmpC-producing *E. coli*, blue striped = MBC NON-ESBL-/AmpC-producing *E. coli*. Arrows mark MIC_95_ and MBC_95_ representing cut-off values for isolates with reduced susceptibility. (**A**) BAC = Benzalkonium chloride, (**B**) DDAC = Didecyldimethylammonium chloride, (**C**) FA = Formaldehyde, (**D**) HP = Hydrogen peroxide, (**E**) PAA = Peracetic acid, (**F**) AA = Acetic acid, (**G**) PCMC = Chlorocresol (p-chloro-m-cresol).

MIC and MBC data showed non-normal, unimodal distributions ranging between one and three dilution steps for all biocides (Figure 2). MIC and/or MBC values above MIC_95_/MBC_95_ indicated isolates with reduced susceptibility to the tested biocides.

These biocide-tolerant isolates were found in all barns (barn 1: *n* = 2; barn 2: *n* = 1, barn 3: *n* = 2, barn 4, *n* = 4), and mostly originated from transitions between wall and floor as well as from cracks and crevices in the ground (Table S1). An individual NON-ESBL-/AmpC-producing *E. coli* isolate (ST351) displayed elevated MIC (160 mg/L) and MBC (320 mg/L) values of FA and an elevated MIC of PCMC (1000 mg/L). Furthermore, three NON-ESBL-/AmpC-producing *E. coli* showed either an elevated MIC (160 mg/L, *n* = 2, ST10, ST351) or MBC value (320 mg/L, *n* = 1, ST1818) of FA. Increased MBCs were also detected for PAA (1000 mg/L, *n* = 1, ESBL-producing *E. coli*, ST117) and BAC (80 mg/L, *n* = 4, three NON-ESBL-/AmpC-producing *E. coli*, ST10, ST162, ST429, and one ESBL-producing *E. coli*, ST117) (Figure 1).

### 3.3. Susceptibility to Antibiotics

All isolates were sensitive to carbapenems (ETP, IMI, MERO), COL and TGC. Antibiotic resistance to AMP (100% ESBL-/AmpC-producing *E. coli*, 63% NON-ESBL-/AmpC-producing *E. coli*), SMX (52% ESBL-/AmpC-producing *E. coli*, 36% NON-ESBL-/AmpC-producing *E. coli*), and TMP (28% ESBL-/AmpC-producing *E. coli*, 39% NON-ESBL-/AmpC-producing *E. coli*) were most common in both groups (Figure 1). Thirty-four isolates (37%) were resistant to at least one antibiotic in three or more classes and therefore defined as multidrug-resistant (MDR). Two isolates from barn 2 were resistant to antibiotics in five substance classes including aminoglycosides, ß-lactams, fluoroquinolones, sulfonamides, and tetracyclines.

### 3.4. In Silico Analysis of Determinants Conferring Biocide and Metal Tolerance

**Figure 3:**
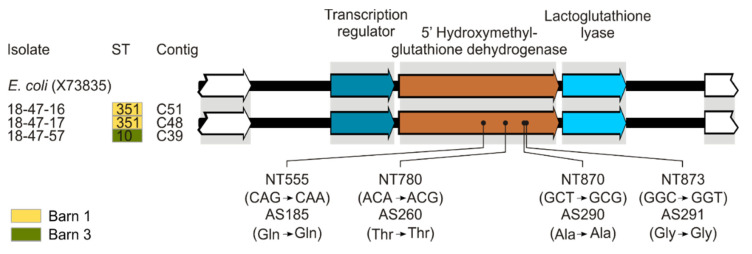
Glutathione-dependent formaldehyde dehydrogenases of *E. coli* isolates compared to the plasmid-encoded reference X73835. The alignment was created using Bionumerics and adjusted by CorelDraw Graphic Suite 3.0 (version 17) for better interpretation. Relevant CDS (arrows) were labeled by protein function based on RAST annotation.

Out of 753 proteins potentially conferring biocide or metal tolerance 249 were identified in our study population (Table S3). Four tolerance determinants were exclusively present in three isolates with increased MIC values of FA (18-47-16 (ST351), 18-47-17 (ST351), and 18-47-57 (ST10). Three of these determinants belonged to an arsenic resistance operon whereas the other one was annotated as nickel/cobalt efflux transporter NcrC that is involved in nickel and cobalt resistance. All isolates under study harbored glutathione- and NAD-dependent formaldehyde dehydrogenase with ≥80% nucleotide identity to the reference (Genbank Acc. No. X73835) found in the formaldehyde-tolerant strain *Escherichia coli* VU3695 [19]. The three isolates with reduced susceptibility to formaldehyde harbored an additional formaldehyde dehydrogenase with 99.6% identity to X73835. Sequence analysis revealed only synonymous mutations compared to the reference (Figure 3).

**Figure 4:**
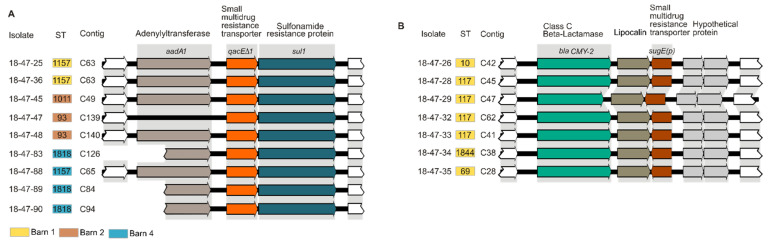
Colocalization of biocide tolerance determinants and antibiotic resistance genes. (**A**) SMR efflux pump encoding gene *qacE∆1* located between aminoglycoside (*aadA1*) and sulfonamide (*sul1*) resistance genes on the same contig. (**B**) SMR efflux pump encoding gene *sugE(p)* located downstream of class C beta-lactamase. The alignment was created using Bionumerics and adjusted by CorelDraw Graphic Suite 3.0 (version 17) for better interpretation. Relevant CDS (arrows) were labeled by protein function based on RAST annotation.

Genes of the *E. coli* acid fitness island were found in all but one isolate of the study population. SMR efflux pump genes *sugE(c)*, *ydgE*, and *ydgF* and the MFS efflux pump gene *mdfA* were always present. We could not detect genes encoding the QAC-specific efflux determinants QacE, QacG, QacF, QacI, and QacH. Seventy-nine isolates (85%) carried *emrE*. The SMR efflux pump gene *qacE∆1* was detected in nine NON-ESBL-/AmpC-producing *E. coli* isolates (10%) of ST93 (*n* = 2), ST1011 (*n* = 1), ST1157 (*n* = 3), and ST1818 (*n* = 3) taken at different sampling sites in the barns 1, 2 and 4 (Figure 4A). *SugE(p)* was detected in seven plasmid-mediated AmpC β-lactamase-(pAmpC-)producing *E. coli* isolates (8%) from barn 1 (ST117 (*n* = 3), ST10 (*n* = 1), ST48 (*n* = 1), ST69 (*n* = 1), ST1844 (*n* = 1)) (Figure 4B). However, the presence of efflux determinants was not associated with reduced susceptibility to tested biocides.

### 3.5. In Silico Analysis of Antibiotic Resistance Gene Profiles

Phenotypic antibiotic resistance could be attributed to known genetic resistance determinants except for gentamicin (Figure 1). Identified determinants responsible for beta-lactam antibiotic resistance were *bla_TEM-1A_* (*n* = 1, ST1157, barn 4), *bla_TEM-1B_* (*n* = 52, 25 STs from all barns), *bla_TEM-1C_* (*n* = 1, ST10, barn 4), *bla_CTX-M-1_* (*n* = 16, ST117, barns 2, 3, and 4) and *bla_CMY-2_* (*n* = 7, ST10, ST48, ST69, ST117, ST1844, barn 1) as well as *ampC* promotor mutations (*n* = 6, ST297, barn 1). Target mutations of *gyrA* (*n* = 20, 10 STs from all barns), *parC* (*n* = 6, ST93, ST162, ST1431, ST1771, ST8132, from all barns) and/or *parE* (*n* = 1, ST1431, barn 4) as well as the resistance genes *qnrB19* (*n* = 3, ST10, ST1011, ST2320, barns 2, 3, and 4) and *qnrS1* (*n* = 3, ST1485, unknown ST, barn 1) were found in quinolone resistant isolates. Chloramphenicol resistance could be attributed to the presence of *cat1* (*n* = 1, ST10, barn 4). All tetracycline resistant isolates were positive for *tet(A)* (*n* = 20, 10 STs from all barns) or *tet(B)* (*n* = 9, ST117, ST162, ST1771, barns 1 and 4). In sulfonamide and trimethoprim resistant isolates the resistance genes *sul1* (*n* = 9, ST93, ST1011, ST1157, ST1818, barns 1, 2, and 4) and *sul2* (*n* = 38, 16 STs from all barns) as well as *drfA1* (*n* = 27, 12 STs from all barns), *drfA5* (*n* = 6, ST58, ST117, ST1431, ST1844, barns 1, 3, and 4), *drfA14* (*n* = 1, ST2320, barn 4) and/or *drfA17* (*n* = 1, ST162, barn 4) were present.

### 3.6. Association Between Reduced Biocide Susceptibility and Antibiotic Resistance and Co-occurrence of Antimicrobial Resistance Genes

Antibiotic resistance was not significantly associated with reduced susceptibility to biocides. There was also no significant difference between isolates from different barns. In addition, ESBL-/AmpC-producing isolates were in general not less susceptible to biocides than NON-ESBL-/AmpC-producing isolates. On the contrary, a higher proportion of NON-ESBL-/AmpC-producing *E. coli* showed reduced susceptibility in terms of MBCs of FA and PCMC compared to ESBL-/AmpC-producing *E. coli* (Figure 2). Interestingly, several isolates carried biocide and metal tolerance genes on mobile genetic elements closely linked to antibiotic resistance genes. For example, eight *qacE∆1*-positive isolates carried *qacE∆1*, *sul1*, and *aadA1* on the same contig (Figure 4A). These determinants could be found downstream of an integron-integrase (*intI*1) gene in four out of nine isolates verifying their localization on a class 1 integron. The same element carried a mercury-resistance operon. Similarly, all *sugE(p)*-positive isolates (*n* = 7) carried *sugE(p)* and *bla_CMY-2_* on the same contig (Figure 4B). Sequence data revealed genes associated with conjugal transfer and transcription in close proximity indicating plasmid localization of *sugE(p)* and *bla_CMY-2_*.

## 4. Discussion

Our study aimed at investigating (i) the phylogenetic diversity and virulence determinants, (ii) potential relationships between susceptibilities to biocides and antibiotics, and (iii) genetic determinants of biocide tolerance and antibiotic resistance of *E. coli* isolates from German broiler fattening farms. The study population consisted of 93 isolates sampled after C&D. Most of the field isolates belonged to phylogroup B1 and F. While phylogroup B1 and A mainly comprise commensals or intestinal pathogens [42], phylogroup F are frequently associated with ExPECs in humans, companion animals, and birds [43,44,45]. Furthermore, ExPEC strains are closely related to avian pathogenic *E. coli* suggesting poultry as a reservoir of zoonotic APEC strains [39,46]. APEC can cause avian colibacillosis, which threatens poultry flocks worldwide. Three of the STs detected in our study, ST10, ST48, and ST117 have been previously linked to APEC strains [47,48,49] and were also isolated from human patients [50,51,52], emphasizing a zoonotic risk. ST297, which is known to be highly prevalent in environmental and food samples, and ST69 were also found in our study population and can be pathogenic for poultry and humans [53]. In general, our data revealed a broad heterogeneity of *E. coli* isolates on German broiler fattening farms with variable numbers of virulence-associated genes involved in adhesion, iron uptake, and cytotoxic activity. ST117 (phylogroup F) and ST429 (phylogroup B2) carried the highest number of iron uptake-related genes. Similarly, Projahn et al. observed a high prevalence of determinants involved in iron acquisition in ST117 isolates collected during the years 2014 and 2015 from German broiler meat production chains [7]. *E. coli* can survive extreme acid stress [54] making use of amino acid-dependent and independent resistance mechanisms [55]. One of the amino acid-dependent systems, encoded by 12 genes of the acid fitness island, is highly conserved in *E. coli* and was found in virtually all isolates of our study population.

*Escherichia coli* can survive hygiene measures, persist over a long period of time, and spread throughout the barns of broiler chicken farms [14,15,56,57]. Overall, phenotypic biocide susceptibility testing did not prove tolerance to disinfectants within our study population since MIC and MBC values of the biocides tested were well below in-use concentrations. Modal MIC values of *E. coli* determined for BAC [58,59,60,61,62], DDAC [63,64], FA [58,60,61], AA [58,65], PAA [66] and PCMC [67,68] in previous studies were similar to our results. In contrast, modal MIC and MBC values of HP reported for avian pathogenic *E. coli* differed by two dilution steps (64 versus 256 mg/L) [61]. So far, breakpoints to distinguish between biocide susceptible and tolerant isolates are missing. Morrissey et al. [29] suggested ECOFFs for the most commonly applied biocides such as BAC, chlorhexidine, triclosan, and sodium hypochlorite considering various species including *E. coli*. According to published MICs (>64 mg/L) and MBCs (>128 mg/L) of BAC, none of our *E. coli* isolates could be defined as tolerant. However, MIC values of biocides are difficult to compare across studies because experimental conditions have not yet been harmonized. In this context, Slipski et al. compared different antimicrobial susceptibility test methods (broth, agar spot colony, and pegged lid biofilms) and showed that the mode of bacterial growth significantly influenced QAC tolerant phenotypes related to SMR over-expression [69]. Thus, standardized methods are urgently needed.

Based on the MIC_95_/MBC_95_ values determined, nine isolates from our study population showed reduced susceptibility to at least one biocide (Figure 1 and Figure 2). Six of these isolates were taken from transitions between floor and wall or cracks and crevices. These are well-known critical locations in broiler houses because they are difficult to clean and disinfect [14,15], and exposure to subinhibitory concentrations of biocides in such niches is very likely. Three out of the nine isolates showed elevated MICs of formaldehyde and one isolate additionally had an elevated MIC of chlorocresol. The most widespread bacterial pathway for formaldehyde detoxification involves a glutathione-dependent dehydrogenase catalyzing the reversible formation of S-formylglutathione and NADH from formaldehyde, glutathione, and NAD [70]. Enzymatic degradation of formaldehyde by a plasmid-encoded variant of the enzyme has been previously described as a formaldehyde resistance mechanism in *E. coli* [19,71,72,73]. In our study, the plasmid-encoded variant of the formaldehyde dehydrogenase was exclusively present in isolates displaying elevated MICs of formaldehyde (160 mg/L) indicating that this enzyme may contribute to the observed phenotype. Interestingly, genes involved in arsenic and nickel/cobalt resistance were also uniquely detected in these formaldehyde tolerant isolates. 

SMR efflux pumps are known to confer resistance to a variety of substances, including QACs and antibiotics [23,24,26,74,75,76,77], and are commonly found in *E. coli* [59,64,78,79]. Since QACs are frequently used for cleaning and disinfection in the food industry, strains armed with appropriate biocide tolerance mechanisms have an increased ability to persist in food processing environments. Not only drugs and toxic metabolites are expelled from bacterial cells by multidrug efflux pumps, molecules that may be important for cell communication, biofilm formation, and osmoregulation or protection of the cell are also released [76,80]. 

In our study, all isolates harbored the putative QAC tolerance conferring genes *sugE(c)*, *ydgE*, *ydgF*, and *mdfA*, while *qacE*, *qacF*, *qacG*, *qacH*, and *qacI* were absent. These results are in line with previous findings on the prevalence of *ydgE/ydgF* (87–100%), *mdfA* (86–100%), and *qac* genes (0–18%) in *E. coli* isolates from different sources [64,79]. The SMR transporters *emrE*, *qacE∆1*, and *sugE(p)* were detected in varying frequencies within our study population. Nevertheless, our data were similar to those obtained from other epidemiological studies on *E. coli* isolated from poultry meat, meat products, and farms in Germany [81], the United States [79], and China [64]. The contribution of *qacE∆1* as a partially functional derivative of *qacE* [82] on QAC tolerance is controversially discussed [83]. As described before [81,84], we were not able to show an association between the presence of *qacE∆1* and reduced QAC susceptibility. The SMR efflux pump SugE has its role in QAC tolerance [26,64] with a rather narrow substrate specificity, including cetyltrimethyl ammonium, cetyldimethyl ammonium, cetylpyridinium, and cetrimide cations [69,74], which may explain the phenotypic susceptibility to BAC and DDAC of isolates carrying *sugE(p)* in our study. 

Antibiotic resistance profiles were generally consistent with zoonoses monitoring data of commensal *E. coli* from broiler fattening farms in Germany, 2016 [85]. However, 8.3% colistin-resistant isolates were reported in the national monitoring program, whereas colistin resistance was not found in our study population. A significant number of isolates showed resistance to three or more classes of antibiotics including critically important antimicrobials as classified by the World Health Organization such as quinolones and 3rd generation cephalosporins [86]. With the exception of gentamicin, all phenotypic resistances could be traced back to genetic determinants. Different mechanisms are known to confer gentamicin resistance. Most common are enzymes modifying the drug by acetylation (aminoglycoside acetyltransferase, AAC), adenylation (adenylate aminoglycoside nucleotidyltransferase, ANT) or phosphorylation (aminoglycoside phosphotransferase, APH) [87,88]. Mutations in the ribosomal target have also been described [89], but could not be confirmed in our isolates. According to clinical breakpoints of CLSI, *E. coli* is supposed to be resistant to gentamicin if MIC ≥ 16 mg/L [90]. As our isolates had MIC values below the clinical but above epidemiological cut-off (ECOFF 2 mg/L), these isolates may have developed resistance. Within the EU, gentamicin is not authorized for use in poultry [91] and resistance is rarely found in conventional broiler stocks in Germany (1.3% in 2016) [85].

In vitro studies showed that antibiotic cross-resistance can occur during bacterial exposure to subinhibitory concentrations of biocides like QACs [92], biguanides [93], and phenolic compounds [94]. The *E. coli* isolates in our study revealed no association between phenotypic biocide tolerance and antibiotic resistance as described before [60,95]. On the contrary, FA and PCMC killed ESBL-/AmpC-producing *E. coli* at slightly lower concentrations than NON-ESBL-/AmpC-producing *E. coli*. Similarly, lower MICs of DDAC were reported for ESBL-/AmpC-producing *E. coli* in another study [81]. 

The biocide tolerance determinants *qacE∆1* and *sugE(p)* were located on mobile genetic elements in close proximity to the antibiotic resistance genes *sul1* and *bla_CMY-2_*, respectively. *QacE∆1* is common in enteric bacteria and is typically associated with the presence of class 1 integrons that carry the sulfonamide resistance determinant *sul1* explaining why all *qacE∆1* positive isolates showed co-resistance to sulfamethoxazole [96]. On the same genetic element, several mercury resistance genes were observed, which frequently occur on plasmids together with antibiotic resistance genes and the *qacE∆1* gene [97]. Furthermore, multiple gene cassettes can be arranged in tandem within these elements conferring additional resistance to ß-lactams, tetracycline, gentamicin as well as aminoglycosides [59,64,79]. Worldwide, *bla_CMY-2_* is associated with pAmpC-producing *E. coli* from poultry [98]. The genetic element, *bla_CMY-2_-blc-sugE*, has already been found in IncK plasmids of *E. coli* isolated from humans in Spain and poultry in Norway and Switzerland [99,100,101]. Plasmids carrying *sugE(p)* and *bla_CMY-2_* antibiotic resistance genes have been detected in various STs of *E. coli* [99,101,102] and may be spread by conjugative transfer to different reservoirs. Even though isolates carrying *qacE∆1* or *sugE(p)* did not show reduced susceptibility to the QACs investigated in our study, the use of QACs in broiler fattening farms may provide selection pressure to strains that carry genes encoding resistance to clinically important antibiotics [64]. 

## 5. Conclusions

Our study revealed a high genetic diversity of *E. coli* isolates from German broiler fattening farms including genotypes characteristic of ExPEC strains. Our findings support the hypothesis that poultry farm environments may act as a reservoir of human ExPEC and could play a role in the spread of facultative pathogenic *E. coli*. While the overall prevalence of biocide tolerant strains was low, the detection of isolates carrying formaldehyde tolerance determinants and at the same time showing a reduced MIC to the compound indicates that the use of disinfectants could have provided selection pressure. The QAC tolerance determinants *qacE∆1* and *sugE(p)* were both located on mobile genetic elements in close proximity to antibiotic resistance genes. In this case, disinfectants may simultaneously select strains with acquired resistance to other antimicrobials. Whether disinfectants can be a driver of antibiotic resistance in zoonotic pathogens from stable to table has to be clarified to assess the consumer risks related to hygiene measures.

## Data Availability

Data is contained within the article or Supplementary Materials.

## Supplementary Materials

The following are available online at https://www.mdpi.com/2076-2607/9/3/651/s1, Table S1: Supplementary information of investigated isolates, Table S2: Biocide tolerance genes and virulence-associated genes (VAGs) screened in this study, Table S3: In silico analysis of determinants conferring biocide tolerance.
