# Supplementary material for: Genetic but No Phenotypic Associations between Biocide Tolerance and Antibiotic Resistance in Escherichia coli from German Broiler Fattening Farms
Source: Microorganisms. 2021 Mar 21;9(3):651. doi: 10.3390/microorganisms9030651 (PMC8003927; doi:10.3390/microorganisms9030651)
Supplement: Supplementary file 1 [file microorganisms-09-00651-s001.zip › microorganisms-1146078-SI/Table S2.pdf]

Supplemental Table S2

Biocide tolerance genes screened in this study

Gene	Gene description	Accession no.	Source
qacEΔ1	quaternary ammonium compound efflux SMR transporter	JN596280	(1)
qacE	quaternary ammonium compound-resistance protein	X68232	(1)
qacF	quaternary ammonium compound efflux SMR transporter	JN596279	(1)
qacH	quaternary ammonium compound resistance protein	FJ160769	(1)
qacI	quaternary ammonium compound resistance protein	HQ875011	(1)
qacG	quaternary ammonium compound resistance protein	FJ950725	(1)
emrE	efflux-multidrug resistance protein	AIGY01000024	(1)
mdfa	multidrug efflux pump/Na(+):H(+) antiporter/K(+):H(+) antiporter	Y08743	(1)
sugE(c)	SMR family transporter	X69949	(1)
sugE(p)	SMR family transporter	HQ023864	(1)
ydgE	multidrug transporter subunit	NC_011745	(1)
ydgF	multidrug transporter subunit	NC_011745	(1)
BW690_25775	S-(hydroxymethyl)glutathione dehydrogenase	X73835.1	(2)
slp (Acid Fitness Island)	starvation lipoprotein	NC_000913	(3)
yhiF (Acid Fitness Island)	putative DNA-binding transcriptional regulator	NC_000913	(3)
yhiD (Acid Fitness Island)	inner membrane protein	NC_000913	(3)
hdeB (Acid Fitness Island)	periplasmic acid stress chaperone	NC_000913	(3)
hdeA (Acid Fitness Island)	acid stress chaperone	NC_000913	(3)
hdeD (Acid Fitness Island)	acid-resistance membrane protein	NC_000913	(3)
gadE (Acid Fitness Island)	DNA-binding transcriptional activator	NC_000913	(3)
yhiU (Acid Fitness Island)	multidrug efflux pump membrane fusion protein	NC_000913	(3)
yhiV (Acid Fitness Island)	multidrug efflux pump RND permease	NC_000913	(3)
gadW (Acid Fitness Island)	DNA-binding transcriptional dual regulator	NC_000913	(3)
gadY (Acid Fitness Island)	small regulatory RNA	NC_000913	(3)
gadX (Acid Fitness Island)	DNA-binding transcriptional dual regulator	NC_000913	(3)
gadA (Acid Fitness Island)	glutamate decarboxylase A	NC_000913	(3)

Virulence associated genes (VAGs) screened in this study

Gene	Gene description	Accession no.	Source	Group
afaB/afaC	afimbrial-adhesin-encoding gene	X76688.1	(4)	adhesion
afaE	afimbrial-adhesin-encoding gene	M12868	(5)	adhesion
bmaE	M-agglutinin subunit	M15677	(4)	adhesion
fimA	type-1 fimbrial protein, A chain	NC_000913.3	(6)	adhesion
fimC	periplasm fimbrial chaperone protein	CP004009.1	(7)	adhesion
fimH	mannose-specific adhesin of type 1 fimbriae	AJ225176	(4)	adhesion
focG	F1C fimbriae subunit	S68237	(4)	adhesion
gafD	G-fimbrial lectin protein	L33969	(4)	adhesion
iha	bifunctional enterobactin receptor/adhesin protein	GU725392	(8)	adhesion
papA	fimbrial major pilin protein	X61239	(4)	adhesion
papC	fimbrial major pilin protein	X61239	(4)	adhesion
papEF	fimbrial major pilin protein	X61239	(4)	adhesion
papG allele I	fimbrial major pilin protein	X61239	(4)	adhesion
papG allele I'	fimbrial major pilin protein	X61239	(4)	adhesion
papG-allele-II	fimbrial major pilin protein	M20181	(4)	adhesion
sfa/foc	S and F1C fimbriae subunits	DQ301498	(4)	adhesion
sfaS	S fimbriae minor subunit SfaS	CP000243	(4)	adhesion
tsh	temperature-sensitive hemagglutinin	AY545598/ AF218073	(9), (10)	adhesion
chuA	outer membrane hemin receptor	U67920.1	(11)	iron uptake
feoB	ferrous iron transporter, protein B	GU361604.1	(12)	iron uptake
fyuA	yersiniabactin/pesticin outer membrane receptor	Z38064	(13)	iron uptake
ireA	iron-responsive element	AE014075	(8)	iron uptake
iroD	salmochelin siderophore system, ferric enterochelin esterase	DQ381420.1	(14)	iron uptake
iroN	iron outer membrane receptor	AF449498	(14)	iron uptake
irp-2	yersiniabactin biosynthetic protein	L18881.1	(15)	iron uptake
iucA	N(2)-citryl-N(6)-acetyl-N(6)-hydroxylysine synthase	X76100.1	-	iron uptake
iucD	Iron uptake chelate protein D	M18968.1	(16)	iron uptake
iutA	aerobactin receptor	X05874	(4)	iron uptake
sitA	structural injection transglycosylase	AY126440.1	(17)	iron uptake
flicC (H7)	H7 variant of the Escherichia coli flagellin gene	NC002695	(8)	miscellaneous
ibeA	invasion of brain endothelium	AF289032	(4)	miscellaneous
ompT	Protease 7	41044	(8)	miscellaneous
PAI(malX)	pathogenicity islands (PAIs)	AF00372	(4)	miscellaneous
cvi-cvaC	colicin V immunity protein-colicin V synthesis protein	X57525	(18)	protectins
iss	increased serum survival protein	CP001855	(8)	protectins
kpsMT (K1)	KI capsular polysaccharide	M57382.1	(4)	protectins
kpsMT II	group 2 capsular polysaccharide units	X53819.1	(4)	protectins
kpsMT III	Group III capsular polysaccharides	AF007777.1	(4)	protectins
neuC	UDP–N-acetylglucosamine (GlcNAc) 2-epimerase	M84026.1	(19)	protectins

ompA	outer membrane protein A	CP004009.1	(7)	protectins
rfc	Escherichia coli O antigen polymerase gene	U39042	(4)	protectins
astA	arginine succinyltransferase	AY545598	(10)	toxins
cdtB	cytolethal distending toxin protein	AJ508930	(20)	toxins
cnf-1	cytotoxic necrotizing factor	U42629	(4)	toxins
hlyA	hemolysin A	M10133	(21)	toxins
hlyD	hemolysin D	2128	(8)	toxins
hlyE	hemolysin E	AF052225	(22)	toxins
hlyF	hemolysin F	14615	-	toxins
vat	vacuolating autotransporter toxin	X16664	(23)	toxins

References

1. Zou L, Meng J, McDermott PF, Wang F, Yang Q, Cao G, Hoffmann M, Zhao S. 2014. Presence of disinfectant resistance genes in *Escherichia coli* isolated from retail meats in the USA. J Antimicrob Chemother 69:2644-9.
2. Kummerle N, Feucht HH, Kaulfers PM. 1996. Plasmid-mediated formaldehyde resistance in *Escherichia coli*: characterization of resistance gene. Antimicrob Agents Chemother 40:2276-9.
3. Mates AK, Sayed AK, Foster JW. 2007. Products of the *Escherichia coli* acid fitness island attenuate metabolite stress at extremely low pH and mediate a cell density-dependent acid resistance. J Bacteriol 189:2759-68.
4. Johnson JR, Stell AL. 2000. Extended virulence genotypes of *Escherichia coli* strains from patients with urosepsis in relation to phylogeny and host compromise. J Infect Dis 181:261-72.
5. Labigne-Roussel A, Schmidt MA, Walz W, Falkow S. 1985. Genetic organization of the afimbrial adhesin operon and nucleotide sequence from a uropathogenic *Escherichia coli* gene encoding an afimbrial adhesin. J Bacteriol 162:1285-92.
6. Riley M, Abe T, Arnaud MB, Berlyn MK, Blattner FR, Chaudhuri RR, Glasner JD, Horiuchi T, Keseler IM, Kosuge T, Mori H, Perna NT, Plunkett G, 3rd, Rudd KE, Serres MH, Thomas GH, Thomson NR, Wishart D, Wanner BL. 2006. *Escherichia coli* K-12: a cooperatively developed annotation snapshot--2005. Nucleic Acids Res 34:1-9.
7. Mangiamele P, Nicholson B, Wannemuehler Y, Seemann T, Logue CM, Li G, Tivendale KA, Nolan LK. 2013. Complete genome sequence of the avian pathogenic *Escherichia coli* strain APEC O78. Genome announcements 1:e0002613-e0002613.
8. Johnson JR, Delavari P, Kuskowski M, Stell AL. 2001. Phylogenetic distribution of extraintestinal virulence-associated traits in *Escherichia coli*. J Infect Dis 183:78-88.
9. Dozois CM, Dho-Moulin M, Bree A, Fairbrother JM, Desautels C, Curtiss R, 3rd. 2000. Relationship between the Tsh autotransporter and pathogenicity of avian *Escherichia coli* and localization and analysis of the Tsh genetic region. Infect Immun 68:4145-54.
10. Skyberg JA, Johnson TJ, Johnson JR, Clabots C, Logue CM, Nolan LK. 2006. Acquisition of avian pathogenic *Escherichia coli* plasmids by a commensal *E. coli* isolate enhances its abilities to kill chicken embryos, grow in human urine, and colonize the murine kidney. Infect Immun 74:6287-92.
11. Clermont O, Bonacorsi S, Bingen E. 2000. Rapid and simple determination of the *Escherichia coli* phylogenetic group. Applied and environmental microbiology 66:4555-4558.
12. Mellata M, Ameiss K, Mo H, Curtiss R, 3rd. 2010. Characterization of the contribution to virulence of three large plasmids of avian pathogenic *Escherichia coli* chi7122 (O78:K80:H9). Infection and immunity 78:1528-1541.
13. Rakin A, Urbitsch P, Heesemann J. 1995. Evidence for two evolutionary lineages of highly pathogenic *Yersinia species*. J Bacteriol 177:2292-8.

14. Johnson TJ, Johnson SJ, Nolan LK. 2006. Complete DNA sequence of a ColBM plasmid from avian pathogenic *Escherichia coli* suggests that it evolved from closely related ColV virulence plasmids. Journal of bacteriology 188:5975-5983.
15. Guilvout I, Mercereau-Puijalon O, Bonnefoy S, Pugsley AP, Carniel E. 1993. High-molecular-weight protein 2 of *Yersinia enterocolitica* is homologous to AngR of *Vibrio anguillarum* and belongs to a family of proteins involved in nonribosomal peptide synthesis. Journal of bacteriology 175:5488-5504.
16. Herrero M, de Lorenzo V, Neilands JB. 1988. Nucleotide sequence of the *iucD* gene of the pColV-K30 aerobactin operon and topology of its product studied with *phoA* and *lacZ* gene fusions. J Bacteriol 170:56-64.
17. Runyen-Janecky LJ, Reeves SA, Gonzales EG, Payne SM. 2003. Contribution of the *Shigella flexneri* Sit, Iuc, and Feo iron acquisition systems to iron acquisition in vitro and in cultured cells. Infection and immunity 71:1919-1928.
18. Otto BR, van Dooren SJ, Nuijens JH, Luirink J, Oudega B. 1998. Characterization of a hemoglobin protease secreted by the pathogenic *Escherichia coli* strain EB1. J Exp Med 188:1091-1103.
19. Zapata G, Crowley JM, Vann WF. 1992. Sequence and expression of the *Escherichia coli* K1 *neuC* gene product. J Bacteriol 174:315-9.
20. Janka A, Bielaszewska M, Dobrindt U, Greune L, Schmidt MA, Karch H. 2003. Cytolethal distending toxin gene cluster in enterohemorrhagic *Escherichia coli* O157:H- and O157:H7: characterization and evolutionary considerations. Infect Immun 71:3634-8.
21. Felmlee T, Pellett S, Lee EY, Welch RA. 1985. *Escherichia coli* hemolysin is released extracellularly without cleavage of a signal peptide. J Bacteriol 163:88-93.
22. Reingold J, Starr N, Maurer J, Lee MD. 1999. Identification of a new *Escherichia coli* She haemolysin homolog in avian *E. coli*. Vet Microbiol 66:125-34.
23. Parreira VR, Gyles CL. 2003. A novel pathogenicity island integrated adjacent to the thrW tRNA gene of avian pathogenic *Escherichia coli* encodes a vacuolating autotransporter toxin. Infect Immun 71:5087-96.
